# Predicting Pain Trajectories in the One Year Following Breast Cancer Diagnosis—An Observational Study

**DOI:** 10.3390/jcm9061907

**Published:** 2020-06-18

**Authors:** Marion Voute, Véronique Morel, Dominique Joly, Christine Villatte, Elodie Martin, Xavier Durando, Bruno Pereira, Gisèle Pickering

**Affiliations:** 1 CHU Clermont-Ferrand, Inserm, Centre d’Investigation Clinique, CIC Inserm 1405, F-63000 Clermont–Ferrand, France; mgauffier@chu-clermontferrand.fr (M.V.); v_morel@chu-clermontferrand.fr (V.M.); elodie.martin63100@laposte.net (E.M.); 2 CHU Clermont-Ferrand, Centre Jean Perrin, Centre de Lutte contre le Cancer, F-63003 Clermont-Ferrand, France; Dominique.JOLY@clermont.unicancer.fr (D.J.); Christine.VILLATTEDEFIGUEIREDO@clermont.unicancer.fr (C.V.); 3 Division de Recherche Clinique, Délégation Recherche Clinique et Innovation, Centre de Lutte contre le Cancer, Centre Jean Perrin, F-63003 Clermont-Ferrand, France; Xavier.DURANDO@clermont.unicancer.fr; 4 CHU de Clermont-Ferrand, Délégation Recherche Clinique & Innovation, 58 Rue Montalembert, F-63003 Clermont-Ferrand, France; bpereira@chu-clermontferrand.fr; 5 Université Clermont Auvergne, Inserm, Neuro-Dol, F-63000 Clermont-Ferrand, France

**Keywords:** breast cancer, pain trajectories, predictive profile, psychosocial vulnerability

## Abstract

The impact of psychosocial vulnerability on pain in the year following breast cancer diagnosis has been little studied. To identify a score of psychosocial vulnerability (cognitive, emotional, quality of life and precariousness parameters) as a predictor of a pain trajectory, we conducted an observational prospective study and included women with newly diagnosed breast cancer. One year follow-up with 3 visits (day of breast cancer diagnosis; 6 and 12 months) aimed to identify distinct pain-time trajectories. Baseline psychosocial vulnerability was characterized by z-score transformation, a higher score representing a more vulnerable patient. A total of 89 patients were included (59.3 ± 10.7 years). Two trajectories of pain were identified—“Transient Pain trajectory” (TP) (39/89 patients) and “Persistent Pain trajectory” (PP) (50/89). A significant difference of pain over time between trajectories (PP vs. TP at 6 months: 2.23 ± 0.23 vs. 0.27 ± 0.09, *p* < 0.001) was observed. Psychosocial vulnerability showed a large effect size (d, −0.82; 95% CI, −1.25 to −0.38; *p* < 0.001) and a higher score in “Persistent pain trajectory” (PP vs. TP: 0.12 ± 0.36 vs. −0.14 ± 0.26, *p* < 0.001). A predictive vulnerability marker of pain development is proposed and could be used at cancer diagnosis to orientate the care pathway of patients experiencing breast cancer.

## 1. Introduction

Breast cancer is the leading type of female cancer in developed countries [1]. The burden of disease is significant, with 279,100 new breast cancer cases estimated in 2020 in the United States [2] and cancer treatment is associated with pain, cognition, emotion and quality of life impairment. Acute and chronic pain are present and prevalence rates for persistent pain following breast cancer surgery range from 25% to 60% [3,4]. Publications have focused on the identification of subgroups of patients with pain trajectories in the first post-operative week [5] and up to 6 months after breast cancer surgery [6,7].

Several significant factors associated with pain after breast cancer surgery have been identified, including preoperative pain intensity, anxiety, opioid consumption, age [8,9,10,11] and psychological distress [12]. Although surgery is the main cause for pain development, patients undergo other interventions including chemotherapy or radiotherapy in a succession that depends on the clinical situation. These interventions do also generate pain, neuropathic pain occurring in 58% patients with cancer chemotherapy [13,14] and moderate-to-severe symptoms of anxiety, sleep disturbances, depressed mood or fatigue co-occurring during cancer treatment [15]. Moreover, it has been shown that cancer, surgery and chemotherapy do induce cognitive function disorders in 16–75% patients [16] and negatively impact memory, executive function, attention and concentration [17,18,19]. Cognitive-emotional status may indeed influence the perception and intensity of pain [20,21,22,23]. Several studies on cognitive and emotional factors such as anxiety, catastrophism, memory and flexibility may characterize the psychological vulnerability of a patient and may predict pain [24,25,26,27]. Social inequalities or precariousness, are also known to have an impact on pain [28,29]. According to the literature and the biopsychosocial aspects of pain, we chose these specific dimensions of psychosocial vulnerability, namely cognitive and emotional function, quality of life and precariousness in this study.

How psychosocial vulnerability before starting breast cancer treatment may have an impact on the development of chronic pain in the year following has not been studied so far in the literature. Therefore, the aim of this study was to describe pain trajectories starting from breast cancer diagnosis and for one year, taking into account all interventions, identifying associated factors (cognitive, emotional, quality of life and social status) and proposing a psychosocial vulnerability predictive score for the development of chronic pain.

## 2. Methods

### 2.1. Study Design

A prospective observational study CanoPEe (Cancer Chronic Pain predicted by Emotional and Cognitive status) was conducted in women with newly diagnosed breast cancer at Jean Perrin Oncology Center, Clermont-Ferrand, France. They were recruited in 2016 with last visit of last patient in 2018. The study was coordinated by the Clinical Research Center (CIC Inserm 1405), University Hospital of Clermont-Ferrand and Ethics approval was provided by the regional Ethics committee, CPP Sud-Est VI (leading ethics committee number AU 895) and registered at “http://www.clinicaltrials.gov” (NCT02777697). A non-opposition form was signed by all participants.

Three visits were scheduled—at the time of cancer diagnosis (baseline), at 6 months (M6) and 12 months after baseline (M12). Patients were also contacted by phone before and after every INT. The first intervention (INT1) was carried out at Day 17 (median (Q1: 13, Q3: 22)), post-INT1 pain was also included in the trajectory analysis. Demographic characteristics were collected at baseline. Pain intensity was recorded at every visit and phone contact in order to identify pain trajectories underlying distinct pain phenotypes. Cognitive, emotional, quality of life and precariousness parameters were recorded at baseline to characterize psychosocial vulnerability score and also at 6 and 12 months.

### 2.2. Participants

Physicians referred women with recently diagnosed breast cancer requiring one or more therapeutic interventions (INT), namely chemotherapy, surgery, hormonotherapy, radiotherapy and/or targeted therapy. Persons with a past history of cancer were excluded.

#### 2.2.1. Pain Measurement

Pain was assessed by a 0–10 Numerical Pain Rating Scale where “0” means no pain and “10” the worst pain possible. “Average pain,” “worst pain” and “pain at the time of call” were collected. The Douleur Neuropathique 4 (DN4) is a screening questionnaire developed to assess the presence of Neuropathic Pain (NP). It consists of 10 items related on the pain description (burning, painful cold, electric shocks) and its associated abnormal sensations (tingling, pins and needles, numbness, itching) and brief bedside neurological examination in the painful area (touch hypoesthesia, pinprick hypoesthesia, tactile dynamic allodynia). For scoring, “1” is given to each positive and “0” to each negative item (total score range 0–10) with a total score of 4 for a diagnosis of NP [30].

#### 2.2.2. Cognitive Function

The Trail Making Test A and B (TMT A/B) examines attention and executive functions in new and non-routine situations. The longer it takes for the patient to finish this test, the more the cognitive functions are diminished [31]. The Functional Assessment of Cancer Therapy COGnitive function (FACT-COG) is a self-report questionnaire that assesses perceived cognitive impairment and consists of 4 subscales (responses ranging from 0 to 4)—“Perceived Cognitive Impairments” (PCI), “impact of perceived cognitive impairments on Quality Of Life” (QOL), “comments from OTHers” (Oth) and “Perceived Cognitive Abilities” (PCA). Higher scores represent better functioning or quality of life [32,33]. The Rey-Taylor Auditory-Verbal Learning Test (RAVLT) is commonly used to assess verbal learning and episodic memory. It consists of presenting a list of 15 words across five consecutive trials. Higher score represents better cognitive function (except for “RAVLT percent forgetting” that is reversed score). Different sub-scores are derived—“RAVLT immediate,” “RAVLT learning,” “RAVLT delayed,” “RAVLT percent forgetting,” “RAVLT true recognition” and “RAVLT learning over trials (LOT)” [34,35]. The Revised Illness Perception Questionnaire (IPQ-R) is a self-questionnaire measuring cognitive illness representations and consists of three sections with several sub-scores. For the identity subscale, patients should circle ‘yes ‘or ‘no’ for each symptom felt related to their current illness. An overall score was calculated by summing all responses. For the causal subscale, patients are asked what they perceive to be the cause of their illness and are asked to respond to each of the listed causes using a five-point Likert-type scale, ranging from 1 (strongly disagree) to 5 (strongly agree). Patients are also asked to rank the 3 most important factors believed to be the cause of their illness. The third section is scored by summing responses to each item is on a five-point Likert-type scale too, ranging from 1 (strongly disagree) to 5 (strongly agree). High scores on the identity, consequences, timeline acute/chronic and cyclical subscales represent strongly held beliefs about the number of symptoms attributed, the negative consequences and the chronicity and cyclical nature of the illness. High scores on the personal and treatment control and coherence subscales represent positive beliefs about controllability and a personal understanding of the illness [36,37,38].

#### 2.2.3. Emotional Function

The Hospital Anxiety and Depression scale (HAD) is a fourteen items self-questionnaire with seven items related to anxiety and seven to depression. Each rated from 0 to 3 with a total score of 21 each. Four classes have been defined: 0–7 = normal, 8–10 = moderate, 11–21 = severe [39]. The Cancer Locus of Control Scale (CLCS), validated in a French population of breast cancer patients, is composed of 17-item disease-specific controllability divided into three subscales. Items are scored on a four-point Likert scale (from 1 “strongly disagree” to 4 “strongly agree”). The items are divided into three subscales—control course and religious control are judged positive for the patient (higher score corresponding to high control) and control cause is related to guilt and blaming oneself (higher score is negative for the patient) [40,41].

#### 2.2.4. Quality of Life and Precariousness

The European Organization for Research and Treatment of Cancer QLQ-C30 (EORTC QLQ-C30) is a questionnaire assessing the quality of life of cancer patients. It is divided in 9 subscales—functional status, symptoms, a global subscale of quality of life and health and six items/isolated symptoms, covering cancer symptoms and frequent side effects of cancer therapies (e.g., loss of appetite). The EORTC QLQ-C30 score ranges from 0 to 100. A high score for a functional scale represents a high/healthy level of functioning, a high score for the global health status represents a high quality of life but a high score for a symptom scale represents a high level of symptomatology/problems [42,43]. The Pittsburg Sleep Quality Index (PSQI) is a questionnaire with nineteen items assessing seven domains—subjective sleep quality, sleep latency, sleep duration, habitual sleep efficiency, sleep disturbances, use of sleep medication and daytime dysfunction. An overall score greater than 5 is an indicator of sleep disorders [44]. The Evaluation of Precarity and Inequalities in Health Examination Centers (EPICES) is a French questionnaire composed of eleven binary questions that assess precariousness related to marital status, health insurance status, economic status, family support and leisure activity. This questionnaire is composed of eleven binary questions relative to dimensions of precariousness ranging from 0 to 100. The higher the score, the more the patient is in a precarious situation (cut-off to categorize people as in precarious situation when EPICES ≥ 30.2) [45].

#### 2.2.5. Psychosocial Vulnerability

In this study, psychosocial vulnerability is defined with a single overall score that encompasses all the z-scores of the questionnaires. This “psychosocial vulnerability score” is then exploited as a predictive marker of chronic pain development in patients with cancer. It was obtained by calculation based on the work proposed by O’Brien [46] concerning composite endpoints—“The average z extends this approach to include continuous, ordinal, dichotomous and time-to-event endpoints. Specifically, continuous, ordinal and dichotomous variables are converted to z-scores by subtracting an individual’s value from the overall mean and dividing by the SD of the pooled group; time-to-event variables are first transformed to log-rank scores and then converted to z-scores by subtracting the mean and dividing by the SD of the pooled data. The z-scores are then aligned to the same direction so that worse outcomes have smaller scores. The z-scores are then averaged across endpoints for each patient” [46].

### 2.3. Statistical Analysis

In order to investigate a predictive dimension in cancer patients on the chronic pain development trajectories after different cancer treatment protocols, sample size estimation was determined sequentially according to rules-of-thumb for determining the minimum number of subjects required to Cohen’s recommendations who has defined effect-size bounds as—small (d = 0.2), medium (d = 0.5) and large (d = 0.8) [47]. So, with a sample size around 100 patients, effect-size greater than 0.8 for comparisons of cognitive-emotional parameters between pain trajectories can be highlighted for a two-sided type I error at 0. and a statistical power greater than 80%.

Statistical analyses were performed using Stata software, Version 13 (StataCorp, College Station, TX, USA). All tests were two-sided, with a Type I error set at 5%. To analyze longitudinal data, random-effects models were performed, with time as fixed effect and patient as random-effect. A Sidak’s type I error correction was applied to perform multiple comparisons. The results were expressed as effect-sizes (d) and 95% confidence intervals. To identify distinctive trajectories of pain group-based trajectory model (GBTM) were carried out to model the relationship between pain and time, for each trajectory, the shape of the trajectory and the estimated proportion of the population belonging to each trajectory. The best-fitting model will be selected according to the Bayesian information criterion (BIC). Then, the continuous variables were compared between groups by Student *t*-test or Mann-Whitney test. The comparisons were carried out using Chi-squared or Fischer’s exact tests. To determine the parameters associated to pain trajectories, multivariable logistic analyses were carried out on covariates fixed according to univariate results and to clinical relevance. A particular attention has been paid to the study of multicollinearity and interactions between covariates. Results were expressed as odds-ratios and 95% confidence intervals.

A sensitivity analysis was carried out to determine the statistical nature of missing data and to apply the most appropriate imputation data approach. GBTM analysis was performed using Last Observation Carry Forward (LOCF) method.

## 3. Results

**Figure 1:**
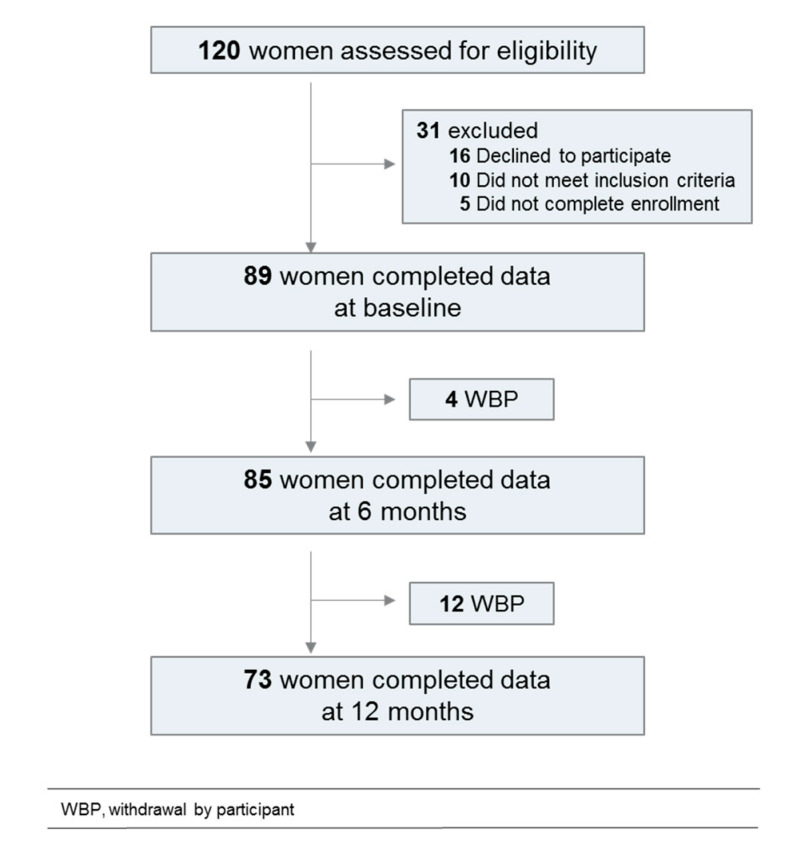
Flowchart of the study population.

The flowchart is shown on Figure 1. Clinical and sociodemographic characteristics are described in Table 1. A total of 120 patients have been prescreened, 89 completed data at baseline, 85 at 6 months and 73 at 12 months. Patients were mostly classified with low-stage tumors. A mean of 3 therapeutic interventions per patient was reported, with surgery, radiotherapy and hormonotherapy in decreasing chronological order.

### 3.1. Longitudinal Analysis

All raw results are grouped in 4 large entities according to questionnaires and tests carried out at each visit—pain measurement, cognitive function, emotional function, quality of life and precariousness. These descriptive data are presented in Supplementary Table S1 corresponding standardized mean difference (effect size) in Supplementary Figure S1.

Pain measurement. We found a significant increase of pain (mean [SEM]) over time compared to baseline (M6 vs. baseline: 0.48 [0.15] vs. 1.62 [0.17], *p* < 0.001; M12 vs. baseline: 0.48 [0.15] vs. 1.41 [0.18], *p* < 0.001). The standardized mean difference was 0.73 (95%CI, 0.52 to 0.94) for M6 vs. baseline and 0.64 (95% CI, 0.41 to 0.84) for M12 vs. baseline, a medium-to-large effect size.

Cognitive function. Regarding the TMT A and B tests, calculated effect sizes were respectively 0.70 (95% CI 0.46 to 0.93) and 0.80 (95%CI 0.53 to 1.07) between baseline and M6 and 0.63 (95% CI 0.40 to 0.87) and 0.72 (95% CI 0.45 to 1.00) between baseline and M12. Two subscores of the FACTCOG questionnaire presented significant results over time—PCI (M6, 0.64, 95%CI 0.43 to 0.86; M12, 0.62, 95%CI 0.39 to 0.85) and PCA (M6, 0.36, 95% CI 0.15 to 0.58; M12, 0.34, 95% CI 0.11 to 0.57). Only one subscore of RAVLT questionnaire showed a standardized mean difference of −0.50 (95% CI −0.87 to −0.12) between baseline and M6 and −0.70 (95% CI −1.17 to −0.12) between baseline and M12. Among the subscores of the IPQR questionnaire, several dimensions described a significant response as “Identity” (M6, 1.46, 95%CI 1.25 to 1.68; M12: 1.23, 95%CI 1.00 to 1.46), “Consequences” (M6, 0.99, 95% CI 0.78 to 1.21; M12, 0.77, 95% CI 0.53 to 1.00), “Personal control” (M6, 0.40, 95%CI 0.18 to 0.61; M12, 0.26, 95%CI 0.03 to 0.49) and “Timeline cyclical” (M6, 0.53, 95%CI 0.31 to 0.75; M12, 0.38, 95% CI 0.14 to 0.61).

Emotional function. Concerning depression, the analysis of the HAD questionnaire indicated a standardized mean difference of 0.92 (95%CI 0.70 to 1.13) between baseline and M6 and 0.67 (95%CI 0.44, 0.90) between baseline and M12, a medium-to-large effect size. The CLCS questionnaire showed a difference for the “control course” subscore of −0.44 (95%CI, −0.65 to −0.22) between baseline and M6 and −0.73 (95%CI −0.96 to −0.29) between baseline and M12.

Quality of life and precariousness. The subscore of quality of life in FACT-COG presented a large effect size on the year of follow-up (M6, 1.29, 95% CI 1.07 to 1.51; M12: 1.15, 95% CI 0.92 to 1.38). Regarding the EORTC QLQ-C30 questionnaire, several subscores presented a medium to large effect size (QOL; physical, role, cognitive and social functioning; fatigue, nausea/vomiting, pain and insomnia). The quality of sleep evaluated by PSQI also described a significate difference (M6, 0.62, 95% CI 0.41, to 0.84; M12, 0.47, 95% CI 0.24 to 0.70). At last, the score about precariousness showed effect sizes of 0.76 (95%CI, 0.54 to 0.97) between baseline and M6 and 0.30 (95%CI 0.07 to 0.53) between baseline and M12.

### 3.2. Pain Trajectories

**Figure 2:**
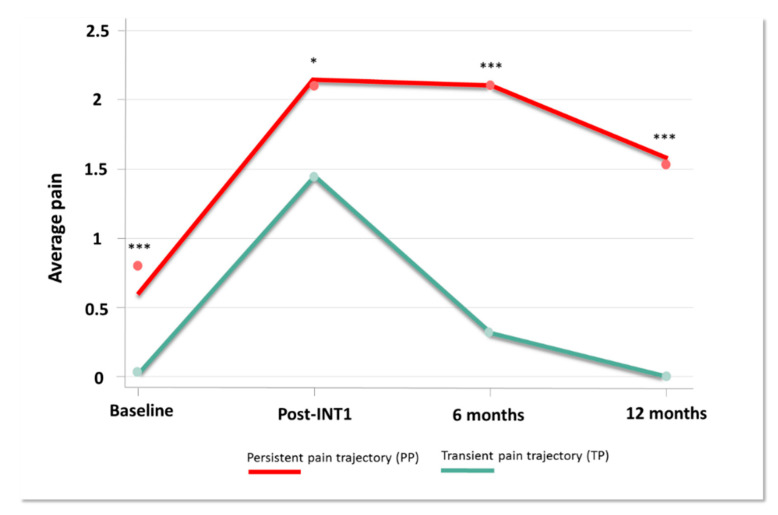
Pain trajectories. Pain intensity (0–10) collected during one year, at the time of cancer diagnosis (Baseline), after the first therapeutic intervention (Post-INT1), 6 months after baseline (6 months) and 12 months after baseline (12 months) (* *p* < 0.05; *** *p* < 0.001).

A two-group model showed the best fit for our data on average pain in breast cancer patients (BIC = 489) with average posterior probabilities equaling 41% and 59% for observed probability of 41.5% and 58.5%, average posterior probabilities at 85.1 and 90.2 (expected >70) and odds of correct classification based on the posterior probabilities of group membership equaling 8 and 6.6 (expected >5) (Figure 2, Supplementary Figure S2) [48]. We identified 2 trajectories of average pain. 1/“Transient Pain trajectory” (TP) included 39 patients (44%) who reported an increased pain intensity after the first intervention. Pain decreased progressively from 6 months and disappeared at 12 months. 2/“Persistent Pain trajectory” (PP) included 50 patients (56%) who showed a larger increase in pain intensity after the first intervention, pain plateauing at 6 months and slightly decreased at 12 months. Results indicated a significant difference on pain intensity between trajectories (mean ± SEM baseline PP vs. TP: 0.86 ± 0.26 vs. 0 ± 0, *p* < 0.001; post-INT1, PP vs. TP: 2.13 ± 0.23 vs. 1.46 ± 0.28, *p* = 0.02; PP vs. TP at 6M: 2.23±0.23 vs. 0.27 ± 0.09, *p* < 0.001; PP vs. TP at 12M: 1.68 ± 0.24 vs. 0 ± 0, *p* < 0.001). Supplementary Table S2 presents patient demographics at baseline. No difference was noted regarding age, medical history, stages, therapeutic intervention (type and number) and presence of neuropathic pain. The distribution of patients with a second INT as well as the delay between INT1 and INT2 does not differ either from one trajectory to another. However, PP trajectory patients had more concomitant treatments (antidepressants, anxiolytics, hypnotics, weak opiate, paracetamol-NSAIDs, antipsychotics, co-analgesics) than the TP trajectory (respectively 32% vs. 3%, *p* < 0.001) with a tendency for a surgery-then-chemotherapy schedule (respectively 35% vs. 13%, *p* = 0.06). Univariate analysis at baseline showed statistically significant differences for several subscores and for z-scores as described in Supplementary Table S3.

### 3.3. Standardized Scores (Z-SCORE)—Psychosocial Vumnerability

**Figure 3:**
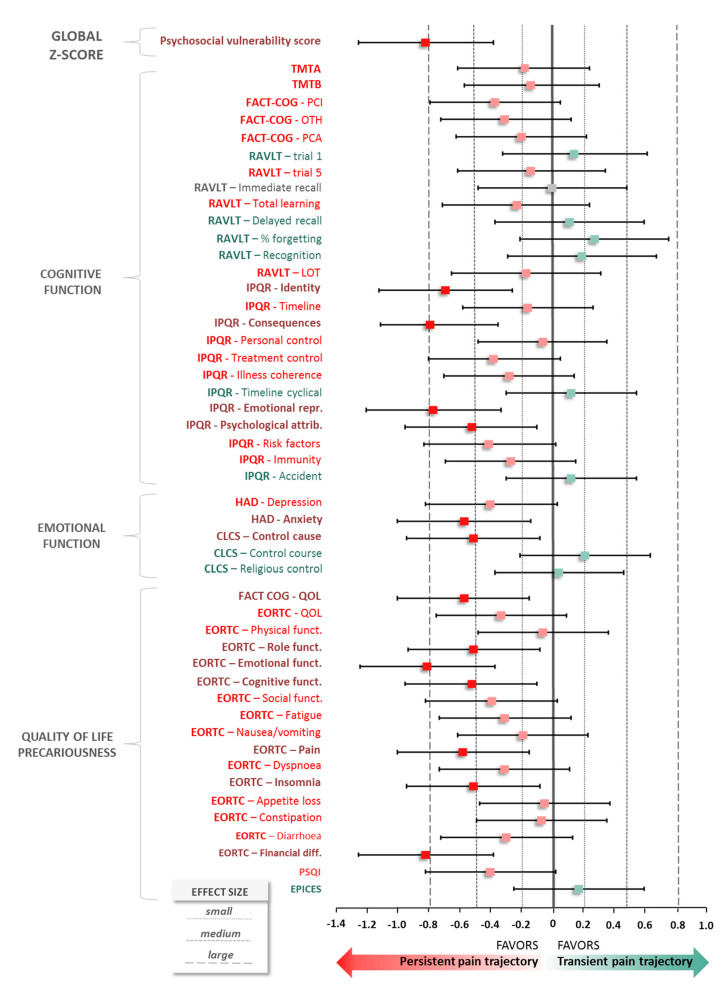
Z-score parameters according to trajectories. Quantitative measure of magnitude of difference of z-score transformation between two pain trajectories: Persistent Pain trajectory (PP) vs. Transient Pain trajectory (TP) and psychosocial vulnerability score. Effect size was calculated as the difference between means of each trajectory divided by standard-deviation, i.e., $\frac{m_{1}-m_{2}}{{\mathrm{SD}}_{\mathrm{pooled}}}=\frac{m_{1}-m_{2}}{\sqrt{\frac{\left(n_{1}-1\right)s_{1}^{2}+\left(n_{2}-1\right)s_{2}^{2}}{n_{1}+n_{2}-2}}}$ with m_1_ and m_2_ the means for each trajectory group, n_1_ and n_2_ the sample sizes and s_1_ and s_2_ the standard-deviations. A negative effect size is indicative of a positive psychosocial vulnerability score (the patient is better), whilst a positive effect size is indicative of a negative psychosocial vulnerability score.

Different pain trajectories were identified in the development of chronic pain. The psychosocial vulnerability of patients was defined at baseline, by analyzing cognitive, emotional, quality of life and precariousness parameters and calculating a predictive score, by z-score transformation. This predictive score showed a significant higher score in the PP trajectory vs. TP trajectory (0.12 ± 0.36 vs. −0.14 ± 0.26, *p* < 0.001), with a large effect size in favor of PP trajectory (d, −0.82; 95% CI, −1.25 to −0.38; *p* < 0.001) (Figure 3). Sensibility analysis concerning GBTM analysis with data imputed using LOCF method highlighted analogous conclusions (d, −0.75; 95% CI, −1.18 to −0.32; *p* = 0.001).

When z-score parameters are independently analyzed, the trend is in favor of PP trajectory for the vast majority of impaired parameters.

Concerning cognitive function, some dimensions of the IPQR questionnaire described a medium-to-large effect size in favor of PP trajectory as “Identity” (d, −0.69; 95%CI, −1.12 to −0.26), “Consequences” (d, −0.79; 95%CI, −1.11 to −0.35), “Emotional representation” (d, −0.77; 95%CI, −1.20 to −0.33) and “Psychological representations” (d, −0.52; 95%CI, −0.95 to −0.10).

Regarding emotion, the analysis of the HAD questionnaire indicated for anxiety a standardized mean difference of −0.57 (95%CI −1.00, −0.14) in favor of PP trajectory. Likewise, the CLCS questionnaire showed a difference for the subscore of “control cause” of −0.51 (95%CI, −0.94 to −0.08).

**Figure 4:**
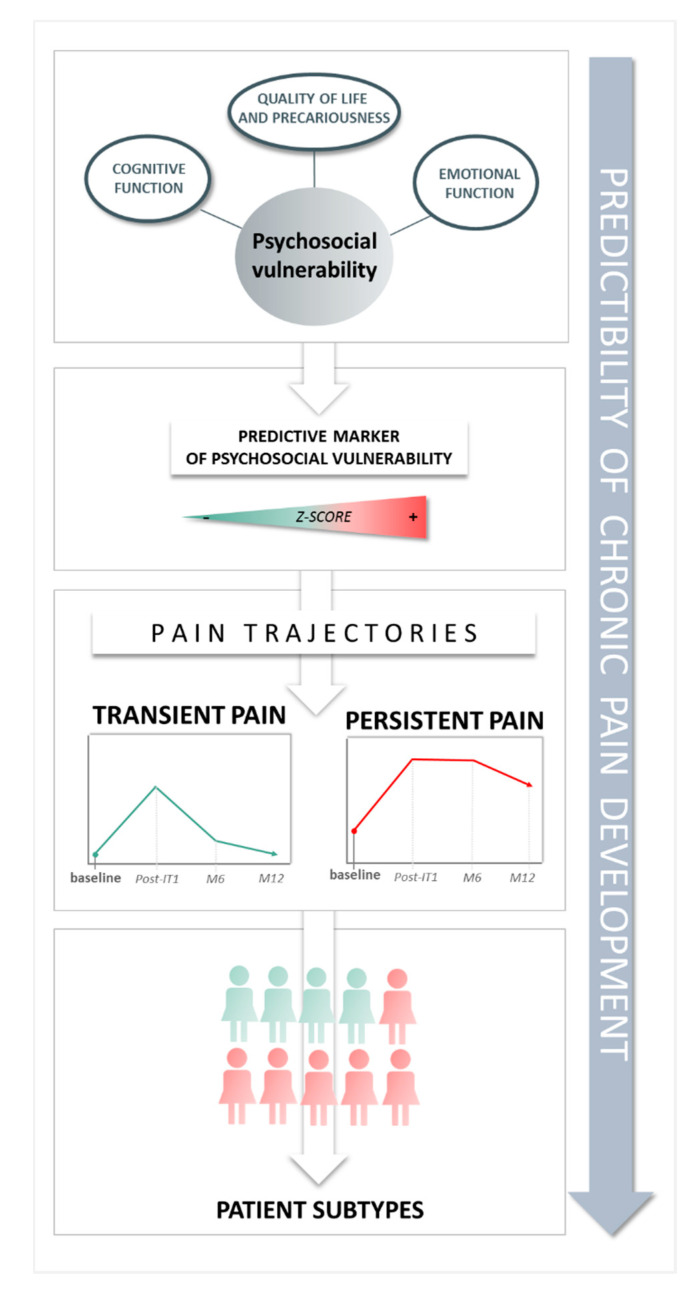
Key findings of the study.

Concerning quality of life, the subscore of quality of life in FACT-COG presented a medium effect size in favor of PP trajectory (d, −0.57; 95%CI, −1.00 to −0.15). Finally, several functional subscores of the EORTC questionnaire were in favor of PP trajectory and described a significant standardized difference as “Role” (d, −0.51; 95%CI, −0.93 to −0.08), “Emotional” (d, −0.81; 95%CI, −1.24 to −0.37), “Cognitive” (d, −0.52; 95%CI, −0.95 to −0.10). Two symptoms on this last questionnaire showed a medium effect size (pain: d, −0.58; 95%CI, −1.00 to −0.15; insomnia: d, −0.51; 95%CI, −0.94 to −0.08). A multivariable analysis is represented as a heatmap in Supplementary Figure S3 and a synthetic figure is proposed in Figure 4.

## 4. Discussion

Our study followed a cohort of patients from their diagnosis of breast cancer and along successive therapeutic interventions, with the aim to identify pain trajectories and a predictive marker of pain development. Pain intensity and other parameters were recorded for one year and this, for the first time, as the literature has so far focused on pain trajectories in breast cancer for shorter periods [5] or on depressive symptoms or sleep quality [49,50,51]. Two pain intensity trajectories have been identified in our study—a TP trajectory (39/89 patients) where women reported a transitional period of pain but were pain-free at 12months and a PP trajectory (50/89) with pain remaining present over time. This persistent pain pattern in 56% of patients confirms published data of 25–60% patients in breast cancer surgery [52,53]. Although pain intensity is mild, as previously described in patients with breast cancer surgery [5], a significant pain increase has been observed at 6 (*p* < 0.001) and 12 months (*p* < 0.001) regardless of the nature and timing of treatments, with a number of patients still with chronic pain after one year.

Beyond the role of different interventions in the development of pain, inherent risk factors [3,24] and psychological vulnerability of the patient are also at play [25,26,27,54].

Our study proposes a predictive psychosocial vulnerability marker for the development of transient or persistent chronic pain in the year following breast cancer diagnosis. This marker combines cognitive, emotional, quality of life and precariousness items and at the time of diagnosis, a larger effect size of the global Z score of these items may predict the patient to belong to a PP trajectory. The analysis of baseline items z-scores for cognition, emotion and quality of life, indicates a number of characteristics of the PP trajectory perspective—negative illness perception [55,56], anxiety [57,58,59,60,61], feeling guilty about cancer [40,62] and poorer quality of life [52,53,63].

The study presents some limitations. First, it is observational with possible associated biases but observational research is valuable in bringing information needed to improve medical decision-making [64]. Another limitation concerns the time required to complete all the questionnaires and the risk of missing data. Finally, the validity of the psychosocial vulnerability score must now be confirmed in an ongoing methodological study with a control population.

Our collective results suggest that patients with breast cancer treatment may follow different pain trajectories, according to their psychosocial vulnerability at the time of diagnosis, with a risk of 59% to follow a “Persistent pain trajectory.” A predictive psychosocial vulnerability marker of pain development has been suggested. Its use at the time of breast cancer diagnosis may orientate the pain and care pathways of cancer patients and contribute to maintain cognitive-emotional functions and quality of life of patients experiencing breast cancer and its treatments. Finally, our data collected in real life now require a methodological validation according to COSMIN guidelines (construct validity, reproducibility and responsiveness) and this could then be extrapolated to other populations in larger cohorts.

## Figures and Tables

**Table 1:** Patient characteristics.

	*n* (%)
Age (mean ± SD)	59.3 ± 10.7
Stages *	
I	19 (23.6)
II	44 (50.6)
III	7 (11.2)
IV	3 (3.4)
Living environment	
Rural	43 (49)
Urban	27 (31)
Semi-urban	18 (20)
Activity	
Inactive	71 (80)
Active	18 (20)
Education	
Low	5 (5)
Medium	62 (70)
High	22 (25)
Medical history	
Locomotor/Rheumatologic	7 (8)
Neurological/Psychiatric	4 (5)
Gynecology	4 (5)
Cardiovascular	2 (2)
ORL	1 (1)
Dermatology	1 (1)
Allergy	1 (1)
Concomitant treatments at baseline (*n* treatment/*n* patient (%))	
Antidepressants	10/9 (10)
Anxiolytics	9/8 (9)
Hypnotics	4/4 (5)
Weak opiate	3/3 (3)
Paracetamol-NSAIDs	2/2 (2)
Antipsychotics	1/1 (1)
Coanalgesics	1/1 (1)
Therapeutic intervention on the year	
Surgery	111 (41)
Radiotherapy	68 (25)
Hormone therapy	57 (21)
Chemotherapy	30 (11)
Target therapy	7 (3)

SD, standard deviation; * 10 missing data.

## Supplementary Materials

The following are available online at https://www.mdpi.com/2077-0383/9/6/1907/s1, Figure S1: Effect size, Figure S2: Pain trajectories after imputation of data, Figure S3: Heatmap z-score, Table S1: Follow-up assessment data, Table S2: Patient demographics at baseline by pain trajectory, Table S3: Patient characteristics at baseline according to pain trajectories.
