# Supplementary material for: Predicting Pain Trajectories in the One Year Following Breast Cancer Diagnosis—An Observational Study
Source: J Clin Med. 2020 Jun 18;9(6):1907. doi: 10.3390/jcm9061907 (PMC7356308; doi:10.3390/jcm9061907)
Supplement: Supplementary file 1 [file jcm-09-01907-s001.pdf]

Supplementary Figure S1. Effect size. Quantitative measure of the magnitude of difference of all scores, the difference was calculated between two time points: at baseline, 6 months and 12 months.

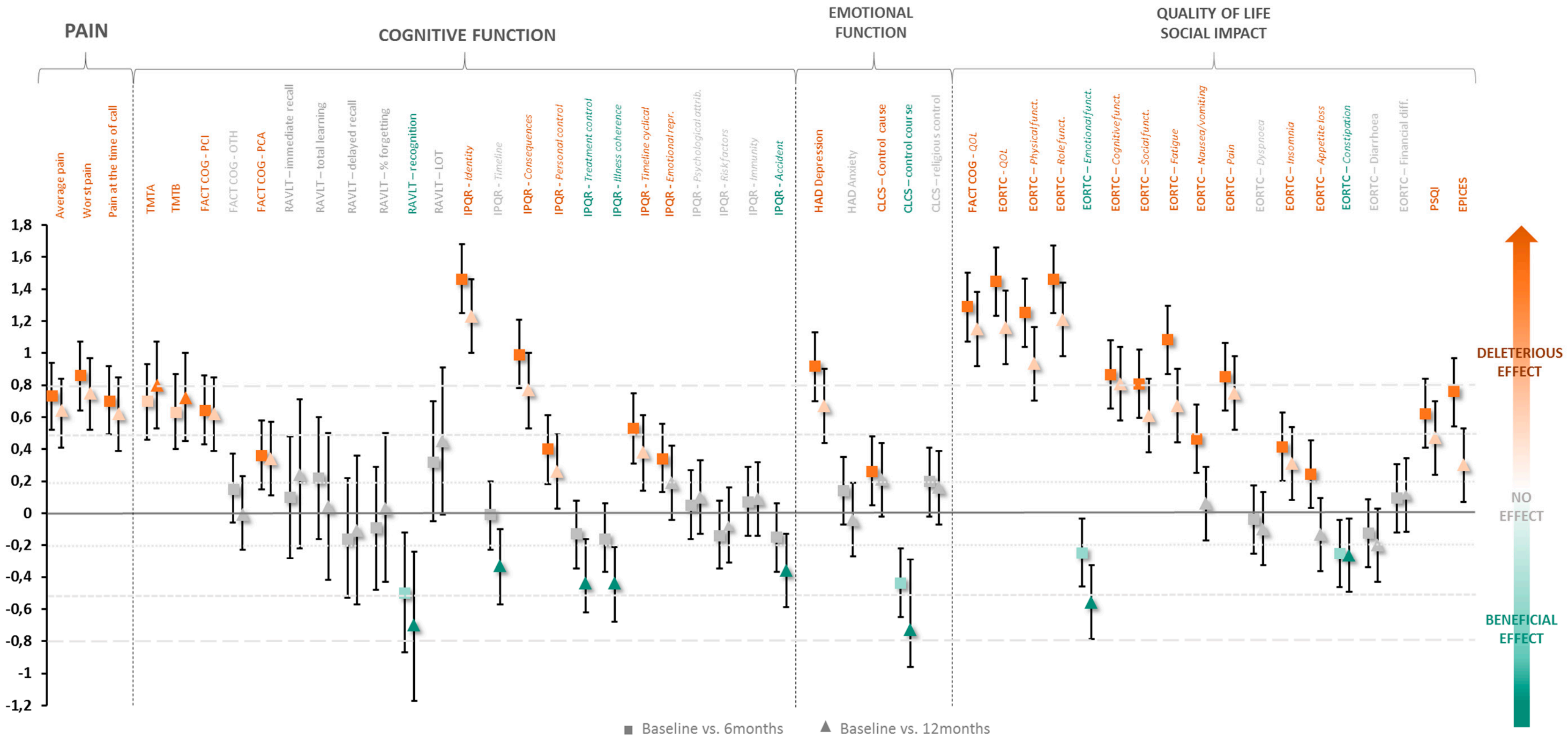

Supplementary Figure S2. Pain trajectories after imputation of data.

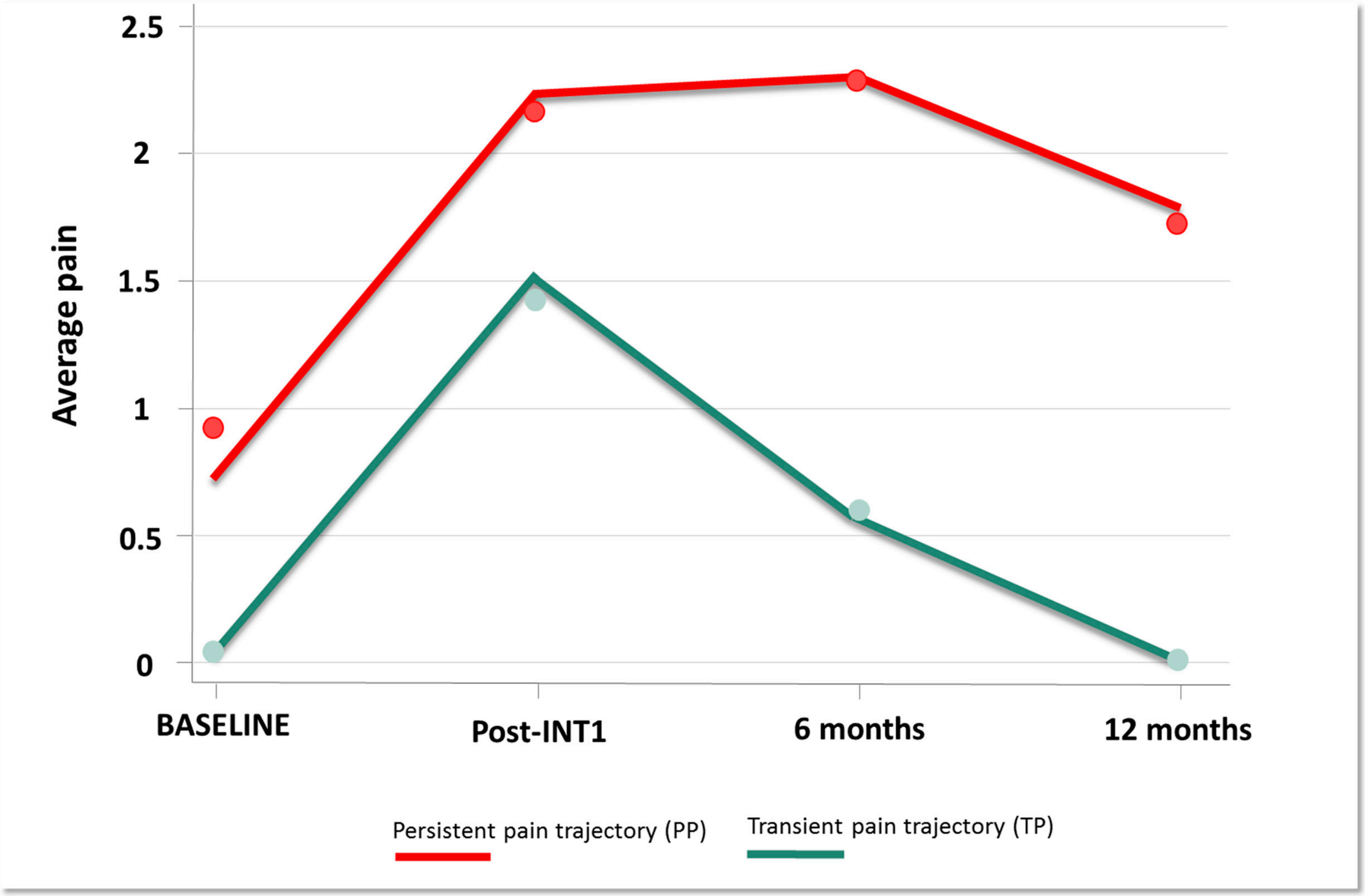

Supplementary Figure S3. Heatmap z-score. Correlation between variables (small correlation: 0.2 to 0.5; medium correlation: >0.5 to 0.7; large correlation: >0.7)

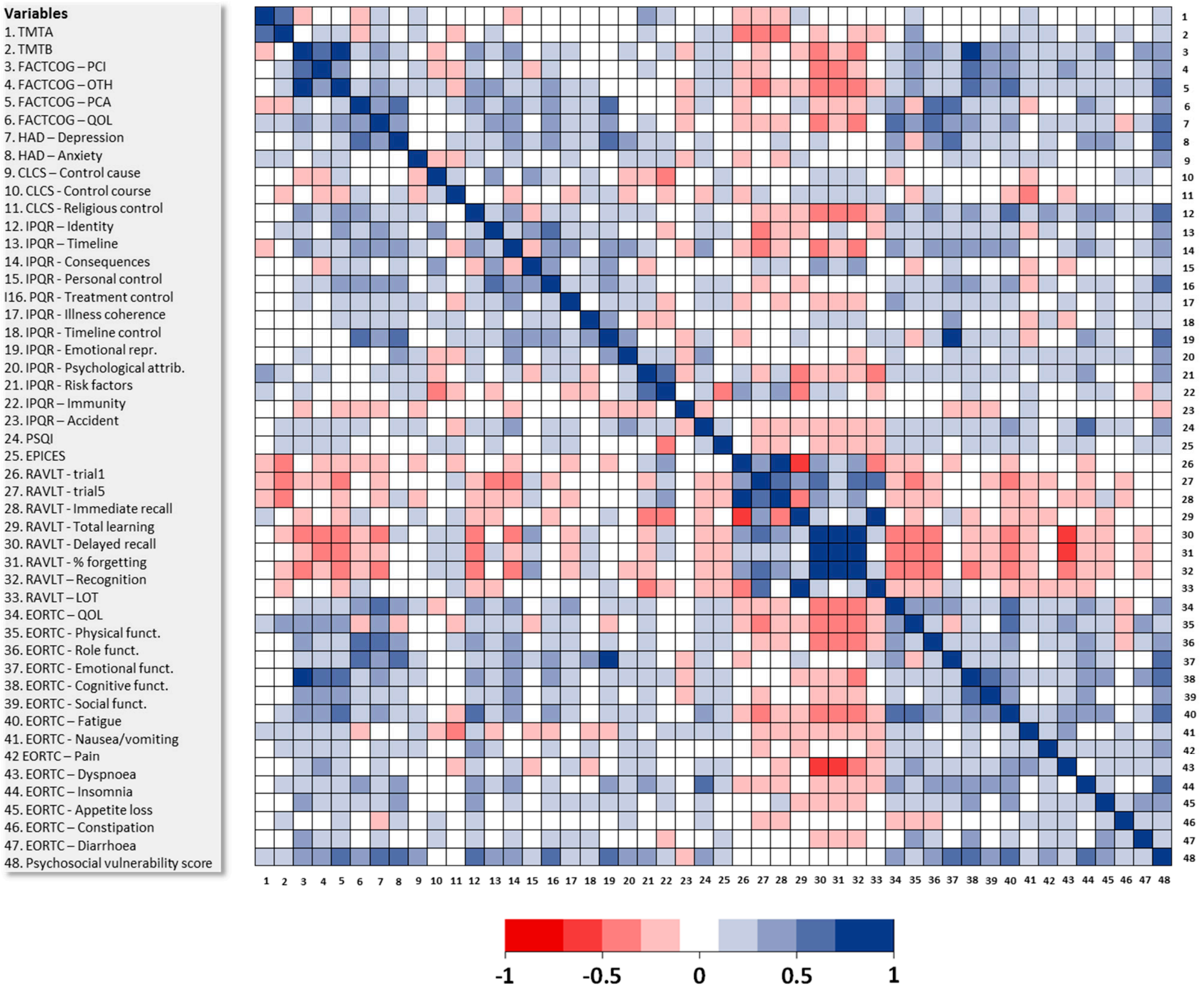

Supplementary Table S1. Follow-up assessment data. All data were collected at baseline, 6 months (6M) and 12 months (12M).

Questionnaires Tests	Sub-score	Baseline	6M	12M	Baseline vs. 6M			Baseline vs. 12M		
		N/mean (SEM)	N/mean (SEM)	N/mean (SEM)	p	ES	[95% CI]	p	ES	[95% CI]
[a]PAIN	*Average pain (-)*	89/0.48 (0.15)	85/1.62 (0.17)	73/1.41 (0.18)	<0.001	0.73	[0.52 ; 0.94]	<0.001	0.64	[0.41 ; 0.84]
	Worst pain (-)	89/0.65 (0.18)	85/2.53 (0.24)	73/2.19 (0.25)	<0.001	0.86	[0.64 ; 1.07]	<0.001	0.75	[0.52 ; 0.97]
	Pain at the time of call (-)	89/0.28 (0.13)	85/1.33 (0.16)	73/1.15 (0.18)	<0.001	0.70	[0.49 ; 0.92]	<0.001	0.62	[0.39 ; 0.85]
[b]TMT A/B	*TMT A (-)*	88/36.83 (1.46)	39/41.41 (1.84)	51/41.12 (2.26)	<0.0001	0.70	[0.46 ; 0.93]	<0.0001	0.80	[0.53 ; 1.07]
	TMT B (-)	83/81.39 (3.63)	69/87.86 (3.89)	50/86.38 (4.80)	<0.0001	0.63	[0.40 ; 0.87]	<0.0001	0.72	[0.45 ; 1.00]
[b]FACTCOG	*PCI (+)*	89/65.27 (1.11)	85/59.15 (1.21)	73/59.85 (1.30)	<0.001	0.64*	[0.43 ; 0.86]	<0.001	0.62*	[0.39 ; 0.85]
	OTH (+)	89/15.47 (0.17)	85/15.21 (0.21)	73/15.53 (0.18)	NS	0.15*	[-0.06 ; 0.37]	NS	-0.01*	[-0.23 ; 0.23]
	PCA (+)	89/20.72 (0.53)	85/19.34 (0.45)	73/19.56 (0.52)	0.001	0.36*	[0.15 ; 0.58]	<0.01	0.34*	[0.11 ; 0.57]
[b]RAVLT	*Trial 1 (+)*	71/6.65 (0.23)	27/7.30 (0.36)	18/7.39 (0.41)	NS	-0.20*	[-0.58 ; 0.17]	NS	-0.21*	[-0.67 ; 0.25]
	Trial 5 (+)	68/14.21 (0.23)	27/14.41 (0.23)	18/14.78 (0.13)	NS	0.08*	[-0.29 ; 0.46]	NS	-0.19*	[-0.65 ; 0.27]
	Immediate learning (+)	68/56.84 (1.20)	27/58.15 (1.51)	18/58.28 (1.18)	NS	0.10*	[-0.28 ; 0.48]	NS	0.24*	[-0.71 ; 0.22]
	Total learning (+)	68/7.56 (0.25)	27/7.11 (0.34)	18/7.39 (0.39)	NS	0.22*	[-0.16 ; 0.60]	NS	0.04*	[-0.42 ; 0.50]
	Delayed recall (+)	68/12.76 (0.41)	26/13.31 (0.35)	18/13.11 (0.52)	NS	-0.16*	[-0.53 ; 0.22]	NS	-0.11*	[-0.57 ; 0.36]
	Percent of forgetting (-)	68/10.73 (2.34)	26/8.92 (2.25)	18/11.39 (3.26)	NS	-0.09	[-0.48 ; 0.29]	NS	-0.03	[-0.43 ; 0.50]
	Recognition (+)	68/13.29 (0.36)	26/14.38 (0.31)	18/14.72 (0.19)	0.01	-0.50*	[-0.87 ; -0.12]	<0.01	-0.70*	[-1.17 ; -0.24]
	LOT (+)	68/23.60 (0.78)	27/21.67 (0.92)	18/21.33 (1.38)	NS	0.32*	[-0.05 ; 0.70]	NS	0.45*	[-0.01 ; 0.91]
[b]IPQR	*Identity (-)*	89/1.61 (0.14)	84/3.94 (0.18)	72/3.46 (0.21)	<0.0001	1.46	[1.25 ; 1.68]	<0.0001	1.23	[1.00 ; 1.46]
	Timeline (-)	89/16.90 (0.27)	83/16.83 (0.30)	72/15.86 (0.37)	NS	-0.01	[-0.23 ; 0.20]	<0.01	-0.33	[-0.57 ; -0.10]
	Consequences (-)	89/16.71 (0.36)	83/19.76 (0.34)	72/18.86 (0.47)	<0.0001	0.99	[0.78 ; 1.21]	<0.0001	0.77	[0.53 ; 1.00]
	Personal control (+)	89/19.58 (0.24)	83/18.86 (0.22)	72/19.11 (0.22)	<0.0001	0.4*	[0.18 ; 0.61]	<0.0001	0.26*	[0.03 ; 0.49]
	Treatment control (+)	89/19.72 (0.21)	83/20.07 (0.28)	72/20.79 (0.36)	NS	-0.13*	[-0.35 ; 0.08]	0.001	-0.39*	[-0.62 ; -0.16]
	Illness coherence (+)	89/19.56 (0.31)	83/20.00 (0.35)	72/20.88 (0.32)	NS	-0.16*	[-0.37 ; 0.06]	<0.0001	-0.44*	[-0.68 ; -0.21]
	Timeline cyclical (-)	89/11.56 (0.28)	83/12.99 (0.26)	72/12.58 (0.27)	<0.0001	0.53	[0.31 ; 0.75]	0.001	0.38	[0.14 ; 0.61]
	Emotional representations (-)	89/20.84 (0.50)	83/21.96 (0.32)	72/21.22 (0.41)	<0.01	0.34	[0.13 ; 0.56]	NS	0.19	[-0.04 ; 0.42]
	Psychological attributions (-)	89/12.78 (0.45)	83/13.04 (0.46)	72/12.89 (0.51)	NS	0.05	[-0.16 ; 0.27]	NS	0.1	[-0.13 ; 0.33]
	Risk factors attributions (-)	89/12.51 (0.37)	83/11.95 (0.33)	72/11.83 (0.35)	NS	-0.14	[-0.35 ; 0.08]	NS	-0.08	[-0.31 ; 0.16]
	Immunity (-)	89/4.70 (0.22)	83/4.78 (0.23)	72/4.69 (0.24)	NS	0.07	[-0.14 ; 0.29]	NS	0.09	[-0.14 ; 0.32]
	Accident (-)	89/4.92 (0.12)	83/4.93 (0.13)	72/4.93 (0.14)	NS	-0.15	[-0.37 ; 0.06]	NS	-0.36	[-0.59 ; -0.13]
[c]HAD	*Depression (-)*	89/2.53 (0.29)	85/5.45 (0.45)	73/4.36 (0.39)	<0.0001	0.92	[0.70 ; 1.13]	<0.0001	0.67	[0.44 ; 0.90]
	Anxiety (-)	89/8.11 (0.45)	85/8.54 (0.40)	73/7.79 (0.38)	NS	0.14	[-0.07 ; 0.35]	NS	-0.04	[-0.27 ; 0.19]
[c]CLCS	*Control cause (-)*	89/13.18 (0.22)	83/13.33 (0.21)	72/13.15 (0.23)	NS	0.26	[0.05 ; 0.48]	NS	0.21	[-0.02 ; 0.44]
	Control course (+)	89/13.07 (0.25)	83/13.75 (0.26)	72/14.15 (0.27)	<0.001	-0.44*	[-0.65 ; -0.22]	<0.001	-0.73*	[-0.96 ; -0.29]
	Religious control (+)	89/3.15 (0.08)	83/3.13 (0.09)	72/3.15 (0.10)	NS	0.20*	[-0.02 ; 0.41]	NS	0.16*	[-0.07 ; 0.39]
[d]FACTCOG	*QOL (+)*	89/9.92 (0.45)	85/5.60 (0.39)	73/6.26 (0.46)	<0.001	1.29*	[1.07 ; 1.51]	<0.001	1.15*	[0.92 ; 1.38]

**[d]EORTC**	*QOL (+)*	89/74.53 (1.63)	85/52.16 (1.35)	73/57.88 (1.44)	<0.0001	1.45*	[1.23 ; 1.66]	<0.0001	1.16*	[0.93 ; 1.39]
	Physical (+)	89/92.86 (1.23)	85/75.61 (1.32)	73/80.64 (1.45)	<0.0001	1.25*	[1.04 ; 1.47]	<0.0001	0.93*	[0.70 ; 1.16]
	Role (+)	89/83.33 (2.47)	85/40.39 (2.82)	73/49.54 (3.10)	<0.0001	1.46*	[1.25 ; 1.67]	<0.0001	1.21*	[0.98 ; 1.44]
**Functional**	*Emotional (+)*	89/69.22 (2.11)	85/73.14 (1.79)	73/78.77 (1.79)	<0.05	-0.25*	[-0.46 ; -0.04]	<0.0001	-0.56*	[-0.79 ; -0.33]
	Cognitive (+)	89/86.52 (2.30)	85/68.43 (2.76)	73/71.00 (3.00)	<0.0001	0.87*	[0.65 ; 1.08]	<0.0001	0.81*	[0.58 ; 1.04]
	Social (+)	89/93.26 (1.61)	85/73.92 (2.73)	73/80.14 (2.79)	<0.0001	0.81*	[0.59 ; 1.02]	<0.0001	0.61*	[0.38 ; 0.84]
	Fatigue (-)	89/20.97 (2.05)	85/43.79 (2.33)	73/33.64 (2.71)	<0.0001	1.08	[0.87 ; 1.29]	<0.0001	0.67	[0.44 ; 0.90]
	Nausea/Vomiting (-)	89/0.19 (0.19)	85/4.31 (1.12)	73/0.68 (0.51)	<0.0001	0.46	[0.25 ; 0.68]	NS	0.06	[-0.17 ; 0.29]
	Pain (-)	89/10.30 (2.02)	85/34.71 (3.13)	73/30.14 (3.45)	<0.0001	0.85	[0.64 ; 1.06]	<0.0001	0.75	[0.52 ; 0.98]
	Dyspnea (-)	89/4.12 (1.49)	85/3.53 (1.37)	73/2.74 (1.26)	NS	-0.04	[-0.25 ; 0.17]	NS	-0.10	[-0.33 ; 0.13]
**Symptom**	*Insomnia (-)*	89/38.20 (3.79)	85/50.98 (3.47)	73/43.84 (3.89)	<0.0001	0.41	[0.20 ; 0.63]	<0.01	0.31	[0.08 ; 0.54]
	Appetite loss (-)	89/5.62 (1.79)	85/10.98 (2.58)	73/2.28 (0.99)	NS	0.24	[0.03 ; 0.46]	NS	-0.13	[-0.36 ; 0.09]
	Constipation (-)	89/8.61 (2.53)	85/3.14 (1.44)	73/3.20 (1.74)	<0.05	-0.25	[-0.47 ; -0.04]	<0.05	-0.26	[-0.49 ; -0.03]
	Diarrhea (-)	89/4.49 (1.78)	85/2.35 (1.34)	73/1.37 (1.37)	NS	-0.13	[-0.34 ; 0.09]	NS	-0.20	[-0.43 ; 0.03]
	Financial difficulties (-)	89/0 (0)	85/0.78 (0.78)	73/0.91 (0.91)	NS	0.09	[-0.12 ; 0.31]	NS	0.11	[-0.12 ; 0.34]
**[d]PSQI**		89/8.12 (0.46)	85/10.07 (0.46)	73/9.18 (0.54)	<0.0001	0.62	[0.41 ; 0.84]	<0.0001	0.47	[0.24 ; 0.70]
**[d]EPICES**		89/12.63 (1.45)	85/19.02 (1.23)	73/14.58 (1.28)	<0.0001	0.76	[0.54 ; 0.97]	<0.01	0.30	[0.07 ; 0.53]

Abbreviations: DN4, douleur neuropathique 4; TMTA/B, trail making test A/B; FACTCOG, functional assessment of cancer therapy cognitive function; PCI, perceived cognitive impairments; QOL, quality of life; PCA, perceived cognitive abilities; RAVLT, rey-taylor auditory-verbal learning test; IPQR, revised illness perception questionnaire; HAD, hospital anxiety and depression scale; CLCS, cancer locus of control scale; EORTC, European organization for research and treatment of cancer; PSQI, pittsburg sleep quality index; EPICES, evaluation of precarity and inequalities in health examination centers; SEM, standard error of means; N, effectif. *reverse score: effect size of 22 subscores have been reversed in order to have the same sense of interpretation (the higher the score, the more negative it is for the patient). [a]Pain; [b]Cognitive function; [c]Emotional function; [d]Quality of life and social impact. Raw scores: (+) higher score are positive for patient; (-) higher score are negative for patient.

Supplementary Table S2. Patient demographics at baseline by pain trajectory.

VARIABLES	TRANSIENT Pain Trajectory			PERSISTENT Pain Trajectory			
	**Total**	**N**	%	Total	N	%	*p*
**Age** *mean[SD]*	39		61.5 [11.6]	50		57.6 [9.6]	ns
**Medical history**	39			50			
Locomotor/Rheumatologic		3	8		4	8	
Neurologic/Psychiatric		0	0		4	8	
Gynecology		1	3		3	6	
Cardiovascular		1	3		1	2	ns
ORL		0	0		1	2	
Dermatology		0	0		1	2	
Allergy		0	0		1	2	
**Concomitant treatment**[a]	39	1	3	50	16	32	<0.001
**Stages**[b]	39			50			
**I**		11	28.2		10	20.0	
**II**		16	41.0		29	58.0	ns
**III**		4	10.3		6	12.0	
**IV**		2	5.1		1	2.0	
**DN4 ≥4**	39	0	0	50	2	4.00	ns
**Therapeutic intervention**[c]	38			48			
Number *mean[SD]*			3.1 [1.1]			3.1 [1.1]	ns
Chemotherapy after 1st surgery		5	13		17	35	0.06

[a]n patient with at least one concomitant treatment; [b]n=10 missing data; [c]n=3 missing data.

Supplementary Table S3. Patient characteristics at baseline according to pain trajectories.

VARIABLES		TRANSIENT Pain Trajectory		PERSISTENT Pain Trajectory		*p*	Z-SCORE	
**Questionnaires/tests**	**Subscores**	**N**	**mean (SEM)**	N	mean (SEM)		ES	[95% IC]
**PAIN**	*Average pain (-)*	39	0 (0)	50	0.86 (0.26)	<0.01	NA	NA
	Worst pain (-)	39	0.03 (0.03)	50	1.14 (0.30)	<0.001	NA	NA
	Pain at the time of call (-)	39	0 (0)	50	0.5 (0.23)	0.03	NA	NA
**DN4>4**	*n (%) (-)*	39	0 (0)	50	2 (4.00)		NA	NA
**GLOBAL Z-SCORE**	*Psychosocial vulnerability (-)*	39	-0.14 (0.04)	50	0.12 (0.05)	<0.001	-0.82	[-1.25; -0.38]
**TMT A/B**	*TMT A (-)*	38	35.39 (2.05)	50	37.92 (2.05)		-0.18	[-0.61; 0.24]
	TMT B (-)	38	78.95 (4.60)	45	83.44 (5.47)		-0.14	[-0.57; 0.30]
**FACTCOG**	*PCI (+)*	39	67.44 (1.11)	51	62.82 (1.87)		-0.37	[-0.79; 0.05]
	OTH (+)	39	15.74 (0.16)	51	15.24 (0.27)		-0.31	[-0.72; 0.12]
	PCA (+)	39	21.28 (0.75)	51	20.10 (0.74)		-0.20	[-0.62; 0.22]
**RAVLT**	*Trial 1 (+)*	37	6.78 (0.32)	34	6.50 (0.35)		0.14	[-0.32; 0.61]
	Trial 5 (+)	36	14.08 (0.35)	32	14.34 (0.30)		-0.14	[-0.61; 0.34]
	Immediate recall (+)	36	56.83 (1.69)	32	56.84 (1.72)		-0.001	[-0.48; 0.48]
	Total learning (+)	36	7.33 (0.35)	32	7.81 (0.35)		-0.23	[-0.71; 0.24]
	Delayed recall (+)	36	12.94 (0.53)	32	12.56 (0.64)		0.11	[-0.37; 0.59]
	Percent of forgetting (-)	36	45.95 (2.77)	32	43.92 (2.65)		0.27	[-0.21; 0.75]
	Recognition (+)	36	13.56 (0.37)	32	13.00 (0.64)		0.19	[-0.29; 0.67]
	LOT (+)	36	23.08 (1.04)	32	24.19 (1.18)		-0.17	[-0.65; 0.31]
**IPQR**	*Identity (-)*	39	1.13 (0.17)	50	1.98 (0.19)	<0.01	-0.69	[-1.12; -0.26]
	Timeline (-)	39	16.67 (0.49)	50	17.08 (0.29)		-0.16	[-0.58; 0.26]
	Consequences (-)	39	15.31 (0.58)	50	17.80 (0.39)	<0.001	-0.79	[-1.11; -0.35]
	Personal control (+)	39	19.67 (0.36)	50	19.52 (0.32)		-0.06	[-0.48; 0.35]
	Treatment control (+)	39	20.13 (0.28)	50	19.40 (0.29)		-0.38	[-0.80; 0.05]
	Illness coherence (+)	39	20.03 (0.44)	50	19.20 (0.43)		-0.28	[-0.70; 0.14]
	Timeline cyclical (-)	39	11.74 (0.34)	50	11.42 (0.43)		0.12	[-0.30; 0.54]
	Emotional representations (-)	39	18.95 (0.13)	50	22.32 (0.08)	<0.001	-0.77	[-1.20; -0.33]
	Psychological attributions (-)	39	11.56 (0.68)	50	13.72 (0.56)	<0.05	-0.52	[-0.95; -0.10]
	Risk factors attributions (-)	39	11.72 (0.48)	50	13.12 (0.53)	0.052	-0.41	[-0.83; 0.02]
	Immunity (-)	39	4.38 (0.33)	50	4.94 (0.29)		-0.27	[-0.69; 0.15]
	Accident (-)	39	5.00 (0.03)	50	4.86 (0.14)		0.12	[-0.30; 0.54]
**HAD**	*Depression (-)*	39	1.92 (0.35)	50	3.00 (0.43)	0.055	-0.40	[-0.82; 0.03]
	Anxiety (-)	39	6.79 (0.69)	50	9.14 (0.57)	<0.05	-0.57	[-1.00; -0.14]
**CLCS**	*Control cause (-)*	39	12.59 (0.39)	50	13.64 (0.32)	<0.05	-0.51	[-0.94; -0.08]

	Control course (+)	39	12.79 (0.13)	50	13.28 (0.10)		0.21	[-0.21; 0.63]
	Religious control (+)	39	3.13 (0.32)	50	3.16 (0.30)		0.04	[-0.37; 0.46]
**FACTCOG**	*QOL (+)*	39	11.26 (0.61)	51	8.98 (0.61)	<0.05	-0.57	[-1.00; -0.15]
**EORTC**	*QOL (+)*	39	77.35 (2.41)	50	72.33 (2.19)		-0.33	[-0.75; 0.09]
**Functional**	*Physical (+)*	39	93.33 (1.51)	50	92.67 (1.86)		-0.06	[-0.48; 0.36]
	Role (+)	39	89.74 (2.95)	50	78.23 (3.64)	<0.05	-0.51	[-0.93; -0.08]
	Emotional (+)	39	77.56 (2.77)	50	62.59 (2.77)	<0.001	-0.81	[-1.24; -0.37]
	Cognitive (+)	39	92.73 (2.65)	50	81.67 (3.41)	<0.05	-0.52	[-0.95; -0.10]
	Social (+)	39	96.58 (1.64)	50	90.67 (2.53)	0.053	-0.39	[-0.82; 0.03]
**Symptom**	*Fatigue (-)*	39	17.66 (2.79)	50	23.56 (2.90)		-0.31	[-0.73; 0.12]
	Nausea/Vomiting (-)	39	0 (0)	50	0.33 (0.33)		-0.19	[-0.61; 0.23]
	Pain (-)	39	4.27 (2.00)	50	15 (3.10)	<0.01	-0.58	[-1.00; -0.15]
	Dyspnea (-)	39	1.71 (1.71)	50	6 (2.27)		-0.31	[-0.73; 0.11]
	Insomnia (-)	39	28.21 (5.81)	50	46.00 (4.75)	<0.05	-0.51	[-0.94; -0.08]
	Appetite loss (-)	39	5.12 (2.61)	50	6.00 (2.46)		-0.05	[-0.47; 0.37]
	Constipation (-)	39	7.69 (3.96)	50	9.33 (3.30)		-0.07	[-0.49; 0.35]
	Diarrhea (-)	39	1.71 (1.71)	50	6.67 (2.86)		-0.30	[-0.72; 0.13]
	Financial difficulties (-)	39	0 (0)	50	0 (0)		0	[0; 0]
**PSQI**	*(-)*	39	7.15 (4.27)	50	8.88 (4.34)		-0.40	[-0.82; 0.02]
**EPICES**	*(-)*	39	13.95 (14.06)	50	11.59 (13.46)		0.17	[-0.25; 0.59]

Raw scores: (+) higher score are positive for patient; (-) higher score are negative for patient / Z-score: the higher the score, the more negative for the patient.
